# Case of Nævus Successfully Treated by the Application of Collodion

**Published:** 1868

**Authors:** L. S. Joynes

**Affiliations:** Professor of Physiology in the Medical College of Virginia


### Case of Nævus Successfully Treated by the Application of Collodion. By L. S. Joynes, M. D., Professor of Physiology in the Medical College of Virginia. (Read before the Richmond Academy of Medicine.)

As every mode of treating surgical diseases, which may supersede the necessity for the performance of painful, dangerous, or disfiguring operations, should be welcomed as a boon to humanity, I feel it a duty to report the following case of the successful employment of a mode of treating vascular tumors, which appears not to be sufficiently known to the profession. The proposition to take advantage of the strong contraction resulting from the evaporation of collodion, in order to effect the obliteration of such tumors, is, I am fully aware, no novelty; but the fact that no mention of such a method is to be found in any of our most approved and recent works on surgery, so far as I have been able to discover, justifies the remark, that it is not generally known to the professian. Yet the results of the occasional trials of it which have been reported, from time to time, afford ground for the belief that it will, in many instances, obviate the necessity of a resort to the ligature, the seton, the irritating injection, the caustic, and other operative procedures, more or less harsh, which the surgeon is accustomed to employ in such cases; while it posesses the great recommendations of causing *no pain*, and leaving *no scar*—advantages especially to be valued, when we reflect that the subjects of these tumors are so generally young children, and that they are so frequently situated on the face.

The subject of the present case was a fine, healthy, female infant, four months old, on whose left cheek there had appeared, two or three weeks before, (according to the report made to me), a small red spot, which gradually enlarged and deepened in color. (I can testify that there was no trace of the disease at birth, for I was present at the birth, and saw the child repeatedly afterwards, until it was five weeks old, when I vaccinated it; during all this time it was "without spot or blemish.") When the case presented itself to me for examination, the spot was about three-quarters of an inch in diameter, slightly elevated above the general surface of the cheek, (of which it occupied the centre,) of a purple hue, offering at a distance some resemblance to a contusion, but on a closer examination, it presented an appearance of vascularity and vitality, quite different from the dull, livid aspect of a contusion. Small veins could be seen through the transparent cuticle creeping over the discolored surface. On examining the part with the fingers, the tumor was found to involve the dermoid and subcutaneous areolar tissue, extending to the limits of the discoloration. It was of softish consistence, without pulsation, and evidently not at all sensitive to pressure.

The case being evidently one of nævus, with a predominance of the capillary and venous elements in its structure, it appeared to me to afford the fairest opportunity for a trial of the virtues of collodion. Accordingly, on the 16th of December, I applied the collodion freely over the discolored surface with a camel's hair pecil. The cantraction which ensued on the drying of the liquid was remarkable. The prominence of the discolored portion of the cheek disappeared; the integument all around was strongly puckered, and through the coating of dried collodion, the discoloration was seen to be much diminished. The application seemed to cause not the slightest pain or inconvenience to the child.

On the next day I re-applied the collodion, and then entrusted the further application to the mother, directing it to be repeated daily, or at least as often as there were any signs of the detachment of the pellicle. I made occasional visits, and found the case progressing favorably.

January 1st. The pellicle having become detached before my visit, while the child was being washed, I found that there was no longer any *tumor* perceptible, either to the eye or the touch; but there still remained a faint discoloration. I therefore continued the treatment.

January 9th. Tnere is no longer any trace of the nævus; the only discoloration visible is due to two or three minute papulæ, produced evidently by the irritating action of the collodion upon the delicate integument. Instead of a prominence, there is now a very slight depression at the site of the nævus, the result of the continued pressure. I directed the application to be suspended for a day or two, in order to allow the subsidence of the slight irritation of the surface, and then to be resumed and continued four or five days longer, in order to confirm the cure.

January 17th. The treatment was discontinued several days ago; the cheek is entirely natural in appearance.

I will add, by way of caution to those who may feel inclined to try this method of treatment, that on one occasion, in applying the collodion, I unthinkingly commenced by making a sweep with the brush around the margin of the tumor, and then rapidly painted over the discolored surface. The result was that the marginal ring dried first, with the effect of producing a partial strangulation of the tumor, which immediately became strongly congested, and much more prominent. The constricting circle, however, was readily dissolved off with a little sulphuric ether, and there was an end of the trouble.

